# Alpha and theta brain activity in 9‐month‐old infants during a live referential gaze paradigm

**DOI:** 10.1111/psyp.14198

**Published:** 2022-10-22

**Authors:** Laura Angelini, Gabriella Tamburro, Francesca Lionetti, Maria Spinelli, Silvia Comani, Filippo Zappasodi, Mirco Fasolo, Tiziana Aureli

**Affiliations:** ^1^ Department of Neuroscience, Imaging and Clinical Sciences Gabriele d'Annunzio University of Chieti‐Pescara Chieti Italy; ^2^ Behavioral Imaging and Neural Dynamics Center Gabriele d'Annunzio University of Chieti–Pescara Chieti Italy; ^3^ Institute for Advanced Biomedical Technologies Gabriele d'Annunzio University of Chieti‐Pescara Chieti Italy

**Keywords:** EEG, ERD/ERS analysis, infants, joint attention, live paradigm, referential gaze

## Abstract

The ability to establish a connection between the direction of the other's gaze and the object that is observed has important implications in the development of social cognition and learning. In this study, we analyzed alpha and theta band oscillations in one group of 9‐month‐old infants by implementing a face‐to‐face live paradigm, which presented the infants with a triadic social interaction with a real human being. We compared neural activations in two experimental conditions: Congruent and Incongruent gaze shift following the appearance of an object. In the Incongruent object‐gaze shift condition, we observed an increase of the theta power in comparison with the Congruent condition. We also found an enhancement of the alpha activity during the Congruent versus the Incongruent object‐gaze condition. These findings confirm the involvement of the theta and alpha band activity in the detection of the gaze of others when it shifts toward a referential target. We consider that the theta band modulation could be associated with the processing of unexpected events. Furthermore, the increase of the alpha band activity during the Congruent object‐gaze condition seems to be in agreement with prior findings on the mechanisms of internally controlled attention that emerge before the first year of life. The implementation of a live paradigm elicited a partially different oscillatory pattern in comparison with non‐live standard paradigms, supporting the importance of an ecological set‐up reproducing real‐life conditions to study the development of social cognition.

## Abstract 2

Our article addresses a key question in the development of social cognition and learning in infants: understanding the neural mechanisms involved in object‐gaze processing. The novel aspect of our work regards the use of a live face‐to‐face paradigm, which could greatly contribute to obtain more reliable information on how infants process referential gaze in a real‐life context.

## 1 INTRODUCTION

One of the milestones of early cognitive development is the infant transition from the ability to engage with another person in dyadic face‐to‐face interactions to the ability to share attention toward the same object/event with another person within “triadic” interactions. Becoming capable of joint attention (JA) (Baron‐Cohen et al., 1995; Bloom, 2002) is foundational for the advancement of abilities as diverse as language acquisition, theory of mind and learning (Brooks & Meltzoff, 2015; Mundy et al., 2007; Tomasello et al., 2005). The impairment of this process is considered one of the earliest indicators of a serious deficit in social cognition development such as the autism spectrum disorder (ASD) (Charman, 2003). In its basic form, JA entails following, driving and monitoring the gaze of a social partner during interaction, and therefore implies the use the other's eye gaze as referential, that is, as a cue that establishes a virtual connection between the direction of the gaze and the object being observed. For these reasons, referential gaze is considered the “touchstone” of JA (Lachat, Conty, et al., 2012). Given that the understanding of referential gaze allows to infer the focus of the people's interest in the environment (Brooks & Meltzoff, 2014), JA is also thought to represent an important precursor of the later higher‐level mental states attributions like perceptions, intentions, interests and desires (Baron‐Cohen et al., 1995), thus playing a pivotal role in the development of social cognition (Bates, 1979; Mundy & Jarrold, 2010).

Infants' looking behavior in response to another person's gaze direction has been the topic of extensive behavioral research since the Scaife and Bruner (1975) seminal finding. Behavioral data showed a developmental progression of the infant's gaze following, from a rudimentary sensitivity to eye gaze direction observed right from birth (Farroni et al., 2004), to a genuine understanding of the referential nature of gaze at about the end of the first year of life (Brooks & Meltzoff, 2005; Woodward, 2003).

More recently, electroencephalographic (EEG) studies added to behavioral data by reporting the brain responses of the infants' object‐gaze processing. Using ERP measurements, Senju et al. (2006) found a posterior face‐sensitive component elicited in 9‐month‐old infants in relation to a gaze shift that was incongruent with the location of a suddenly appearing object as compared to a Congruent gaze shift condition. This effect, similar to what was found in adults, has been ascribed to the mechanism of “violation of expectation” for human actions: when an object appears, people expect that their social partner directs their gaze toward the place where the object appeared; if the partner looks elsewhere, a cognitive re‐organization is needed with a consequent demand for attentional resources and an increased neural processing (Pelphrey et al., 2003, 2005; Senju et al., 2006; Tipples et al., 2013).

To deepen the understanding of the neural mechanisms involved in object‐gaze processing, induced oscillations have been used in addition to ERPs, allowing to gather information about the responses that are not phase‐locked to the temporal onset of the stimulus (Lachat, Hugueville, et al., 2012). All adult studies highlighted a major involvement of the alpha and theta band activities. As to the alpha band, Rayson et al. (2019) obtained similar results in infants aged 6.5–9.5 months, and, in agreement with Michel et al. (2015), they observed a larger suppression of the alpha activity in the central and parietal brain regions when infants observed an adult directing the gaze at the same object that they were looking at, rather than in a different direction. This alpha suppression was more evident in children at 9.5 months than at younger ages, thus providing evidence that, although infants become sensitive to the others' gaze very early (Grossmann et al., 2013), important neural and behavioral developments occur during the second half of the first year of life. Given that both adult (8–13 Hz) and infant (5–8 Hz) alpha‐band oscillations are often designated as “idling rhythms”—given that they correspond to a brain resting state and are attenuated by sensory stimulation—the suppression of the alpha activity when the infants are presented with a gaze‐cued stimulus may be related to the attentive processing elicited by the task (Hoehl et al., 2009; Senju & Johnson, 2009). However, although the parieto‐occipital alpha rhythm is generally associated with arousal mechanisms (Ward, 2003) and social interaction (Foxe & Snyder, 2011; Gale et al., 1972), the modulation of the alpha rhythm amplitude has been also associated with multiple cognitive processes including attention, and its functional significance may vary depending on the task and on the different processing stages (Xie et al., 2018).

A modulation of the theta activity in the frontal brain regions was also found in association with object‐gaze processing. Michel et al. (2015) showed a greater theta synchronization in 5‐month‐old infants for object‐averted with respect to object‐directed eye gaze, similarly to adults' patterns (Berger et al., 2006). Theta synchronization during an Incongruent condition was explained by a mechanism of error detection when the expectation is violated (Conejero et al., 2018; Köster et al., 2019, 2021). Specifically, Bazhenova et al. (2007) speculated on the involvement of an executive attention network during an error prediction processing. This explanation was supported by the results of Orekhova et al. (1999) who observed that, in infants aged 8–11 months, the theta rhythm increased during a peek‐a‐boo game, that requires internally controlled attentive processes. Given that the executive attentional network is thought to mature between 6 and 12 months of age, a theta power increase could be considered as a sign of maturation of the voluntary control of attention.

A methodological issue should be also considered when accounting for EEG data in social cognition research. So far, face and eye gaze processing have been commonly investigated by displaying pictorial stimuli on a computer screen. This procedure is rather different from real human interactions that occur in the everyday environment, which are dynamic events involving a triadic relation between two persons and an object and have a profound affective significance (Hietanen et al., 2020; Pönkänen, Alhoniemi, et al., 2011; Pönkänen, Peltola, & Hietanen, 2011; Saito et al., 2010; Shimada & Hiraki, 2006; Striano et al., 2006). As highlighted in the literature, it is time to start “getting real” in the study of socio‐cognitive processes, namely when studying the processing of eye gaze direction (Kingstone et al., 2003), which has evolved as the main cue for these processes (Lachat, Conty, et al., 2012). Therefore, the functioning of the “social brain” needs to be explored through new ecological study designs, that are close to a natural setting and encompass the presence of a real social agent (Risko et al., 2012). It is worth noting that factors such as the use of dynamic real faces, the physical proximity with another person and the consequent gaze engagement, enhance the personal involvement and elicit patterns of cortical activations that can differ from those elicited by schematic faces or static images, as it occurs in passive interactions based on PC standard paradigms (Pönkänen et al., 2008; Pönkänen, Alhoniemi, et al., 2011; Pönkänen, Peltola, & Hietanen, 2011). Indeed, the modulation of the neural activations depends on the degree of approximation of the real‐life conditions and was ascribed to the effect of the saliency of the context: the greater the social and personal relevance of the stimulus, the greater the level of arousal with consequent higher neural processing. To our knowledge, only one study (Striano et al., 2006) implemented a live paradigm in the field of social cognition development and was used to examine the neural correlates of JA abilities in infants. Striano et al. (2006) reported that the attention of 9‐month‐old infants to a novel object, associated with the amplitude of the Nc component, was enhanced in joint attention conditions where the adult had previously gazed at the infant as it usually happens in the real life. This finding suggests that the eye‐to‐eye contact signaling JA in a live paradigm influenced the neural processing of the objects. Data from this study were subsequently analyzed to assess oscillatory brain activity (Hoehl et al., 2014), and an alpha desynchronization was found when the objects were presented during JA as compared to non‐JA conditions, thus confirming previous ERP results. These findings suggest the importance of assessing the abilities related to social interaction within an ecological setup: given that the degree at which the stimulus approximates real life can differently modulate the patterns of neural activation, live paradigms can aid to advance the understanding of how infants process communicative cues in the real world.

The present study aimed to examine the infant's brain oscillatory activity in response to the other's referential gaze in a live interactive paradigm. To this aim, the non‐live gaze shift paradigm previously proposed by Senju et al. (2006) was modified to provide the infants with a more naturalistic approach that permits to involve them in a face‐to‐face interaction with a real person instead of presenting them with computerized virtual stimuli. We expected to observe gaze cueing effects also in this new experimental setup, but we hypothesized that some differences could be observed in the infant's brain oscillatory activity with respect to standard non‐live situations because of the supposed more arousing live paradigm. Given that the role of a gaze cueing is considered relevant for making social interactions significant, investigating the infant ability to detect the relationship between the looker and the object in a more ecological paradigm could allow to gain new insights on this process.

## 2 MATERIALS AND METHOD

### 2.1 Participants

Fifty‐two 9‐month‐old infants were recruited for participating in the study. Thirty‐five infants were excluded from the analysis for various reasons, such as poor quality of the EEG data or incomplete data recording due to the infant fussiness or their lack of participation in the experiment because of its long duration. This relatively high drop‐out is consistent with what occurred in other EEG studies in infants of similar age (attrition rate can range from 35% to 75%, De Haan, 2007). The study group was then composed of seventeen 9‐month‐old infants (seven females) aged between 254 and 285 days (mean: 273 ± 5 days). All infants were born full term (37–41 weeks) and healthy. The study was approved by the local Ethics Committee and complied with the ethical standards outlined in the declaration of Helsinki. Prior to study participation, the mother of each infant signed the parental informed consent.

### 2.2 Experimental procedure

At their arrival in the laboratory, the general EEG procedures were explained to the mothers, who were told that the experiment concerned face processing and that they had to remain relatively motionless during the experiment. During the experiment the infants were seated on their mothers' lap in a dimly lit, sound‐attenuated and electrically shielded cabin, at a viewing distance of 70 cm from a female experimenter sitting behind a black puppet stage placed in front of the infant. Only the experimenter head and shoulders were visible, with the rest of her body covered by a black panel (size 80 × 70 cm^2^). Behind the experimenter, there was another vertical black panel (160 × 100 cm^2^) with two small holes of 4 × 4 cm^2^ at the experimenter eyes level, one on the left and the other on the right‐hand side of the experimenter. A second experimenter, located behind this vertical black panel, was in charge of giving the timing for the different experimental phases to the first experimenter and of presenting an object (chosen from a set of 60 objects) alternatively at the left or the right hole. The objects were colored toys controlled for size (7 ± 2 cm^2^). During the inter‐trial interval, the infant attention was drawn by a brief adult vocalization. A mirror placed behind the participants' position reflected the first experimenter face and the appearing objects, so that an EEG‐synchronized video‐camera centred at the infant's face could record the infant gaze and behavior, the first experimenter face, and the events occurring during the experiment.

**FIGURE 1:**
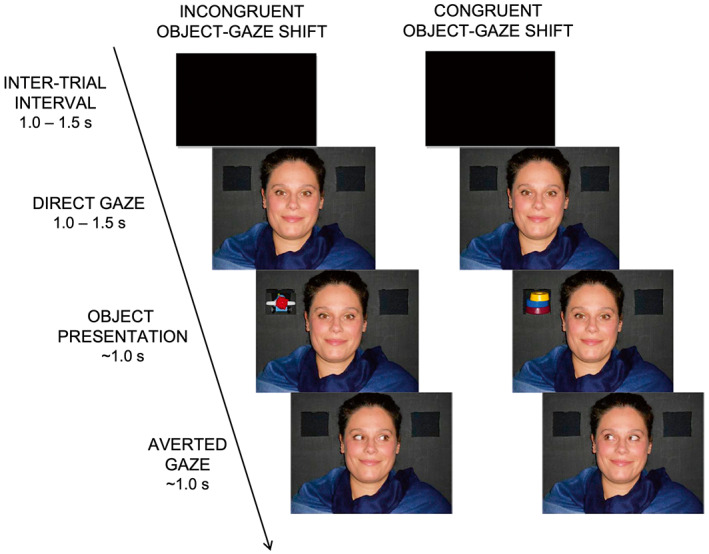
Example of experimental trial for Congruent (right) and Incongruent (left) object‐gaze shift conditions

Each experimental session included 60 trials. Each trial consisted of four phases (Figure 1): inter‐trial interval, direct gaze, object presentation, averted gaze. When the infant was still and attentive, the live procedure started. Each trial started with the first experimenter pulling down the black panel to reveal her face, which displayed a happy emotion, and to gaze straight at the infant (direct gaze phase); her eyes were at the same height of the infant's eyes. After an interval of 1.0–1.5 s, an object was presented at one of the two small holes on the black vertical panel for about 1 s by the second experimenter, who wore black gloves (object presentation phase). During this phase, the first experimenter remained motionless with the same smiling mutual gaze. During the averted gaze phase, which started when the object was removed, the first experimenter—continuing to display a happy emotion—started to move her eyes by 45° to the left or to the right (gaze shift without change in head orientation), that is, to the direction where the object appeared (Congruent object‐gaze shift condition) or to the opposite direction (Incongruent object‐gaze shift condition); the first experimenter kept her gaze in the chosen direction for about 1 s. At the end of the averted gaze phase, the experimenter pulled up the black panel to cover her face for 1.0–1.5 s (inter‐trial phase). The gaze shifts to the left and to the right were balanced across trials in both Congruent and Incongruent object‐gaze shift conditions.

If the infant became fussy or uninterested in the interaction with the experimenter, the experimenter gave the infant a short break. The experiment was interrupted if the infant's attention could no longer be attracted and was not engaging with the experimenter's face.

### 2.3 EEG recordings and analysis

EEG recording of the infant brain activity was performed with a 128 wet electrode net (Electrical Geodesics, version 1.1). Skin‐electrode impedance was measured before the recording and kept below 100 kΩ. EEG data were sampled at 250 Hz and processed off‐line. A video‐camera, with a frame rate of 60 Hz, was used to record both the infant behavior and the face of the experimenter. The video camera and the EEG recording were synchronized by the hardware and software of the Netstation of the EGI system, so that it was always possible to exactly associate the time instant when a video frame was taken with the corresponding time instant of the EEG recording.

Each EEG dataset was filtered between 0.5 and 40 Hz (second‐order Butterworth filter, forward‐backward filtering). Epochs contaminated by head or body movements, saturated or corrupted by instrumental noise, were identified by visual inspection and removed. EEG channels affected by excessive noise were eliminated and substituted by spline interpolation. A semiautomatic independent component analysis–based procedure (Barbati et al., 2004) was applied to identify and remove cardiac and ocular artifacts. Data were considered for the analysis only if the number of corrupted channels was lower than 12 (approximately the 10% of the total EEG sensor number). EEG signals were re‐referenced to the common average reference. A minimum number of eight cleaned trials for each condition was required.

For each trial, the video recording was inspected to assess that the infant kept its gaze at the experimenter for the entire trial duration. Trials for which this condition was not satisfied were excluded from further analysis. Since the paradigm was live and no electronic trigger events were available, the videos were also used to select the time instants to be used as triggers for the different trial phases. Specifically, the first video frame where the experimenter's face appeared was chosen as the onset of the direct gaze phase; the video frame where the object appeared was chosen as the onset of the object presentation phase; the video frame where the experimenter's eyes were moved to the left or to the right served as the onset of the averted gaze phase. Given the live modality of the experiment, it could happen that the first experimenter initiated to move her eyes before the second experimenter had given the start command. For this reason, a control of times was done. The trials in which the interval between the direct gaze phase onset and the object presentation phase onset was shorter than 800 ms were not included in the analysis. Conversely, the averted gaze phase was excluded if the time between the object presentation phase onset and the onset of the gaze shift phase was less than 600 ms. The variability of the time intervals across trials during the live paradigm was calculated as the mean and the standard deviation across subjects, and by the mean across subjects of the variation coefficients (i.e., the standard deviation over the mean of the trials between each single subject). The lower the variation coefficient, the lower the variability across trials.

### 2.4 Time–frequency analysis

The time–frequency representation (TFR) was computed for each EEG channel by means of a continuous Complex Morlet transformation (Tallon‐Baudry et al., 1997) in the frequency band 2–40 Hz, at 1 Hz frequency resolution. The TFR of the signal power was obtained as the squared magnitude of the complex wavelet‐transformed EEG data. Given that the aim of this analysis was to detect possible differences in band power modulation between the Congruent and Incongruent gaze shift conditions of the averted gaze phase, the signal power was averaged separately for the Congruent and Incongruent conditions in signal epochs of 1500 ms, starting from 500 ms before the gaze shift onset until 1000 ms after the gaze shift onset. Then, the averaged signal power for the Congruent and Incongruent conditions was rescaled in order to show changes relative to the corresponding baseline period and expressed as the percentage of this baseline power. Similarly to Senju et al. (2006), we chose an interval of 400 ms immediately before the beginning of the object presentation phase to calculate the baseline for the ERD/ERS analysis. This means that the baseline was chosen in the final part of the time interval corresponding to the direct gaze phase.

The time–frequency ranges for statistical analyses were chosen by visual inspection on the grand‐average of the time‐frequency plots and according to previous studies where similar experimental paradigms or frequency analysis in infants of the same age of our experimental group were used (Michel et al., 2015). Specifically, in 9‐month‐old infants, the theta activity in the frontal brain areas was more evident in the 4–5 Hz frequency range, whereas the alpha activity was observed in posterior brain areas mainly in the 6–8 Hz frequency range, modulated by stimulation (Marshall et al., 2002; Michel et al., 2015; Saby & Marshall, 2012). The values of the relative power of each EEG channel were averaged in the theta (4–5 Hz) and alpha (6–8 Hz) bands in the time window of 300–800 ms after the onset of the averted gaze phase, for both Congruent and Incongruent object‐gaze shift conditions.

### 2.5 Statistical analysis

**FIGURE 2:**
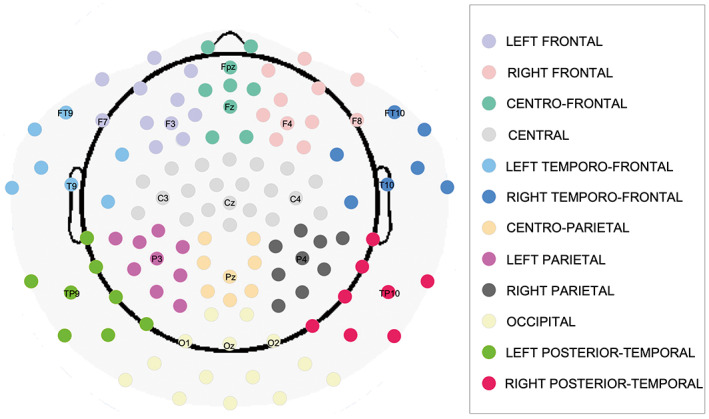
Layout of the EEG net. EEG sensors are colored according to the Regions of Interest defined for the analysis. Twenty‐one standard locations of 10–20 international system are indicated

Theta and alpha relative power values of the EEG channels were grouped in regions of interest (ROIs), similarly to what done by Xie et al. (2018). Specifically, we defined 12 ROIs (Figure 2): left frontal, right frontal, centro‐frontal, central, left temporo‐frontal, right temporo‐frontal, centro‐parietal, left parietal, right parietal, occipital, left posterior‐temporal, right posterior‐temporal. The theta and alpha relative powers of the channels of each ROI were averaged. These averaged relative power values served as the dependent variable for the statistical analysis. Normal distribution was assessed by Kolmogorov–Smirnov test.

A 12 × 2 repeated measure analysis of variance (ANOVA) model was separately applied to the theta and alpha values, with *ROI* and *Condition* (Congruent and Incongruent object‐gaze shift conditions) as within‐subject factor. Partial eta‐squared ($\eta_{P}^{2}$) was used as a measure of the effect size: $\eta_{P}^{2}$ = 0.01 is considered a small effect, $\eta_{P}^{2}$ = 0.06 is considered a medium effect, and $\eta_{P}^{2}$ > 0.14 is considered a large effect. When an interaction *ROI* × *Condition* was found, a paired *t* test was applied to the theta or alpha relative power values to assess the differences between the Congruent and Incongruent object‐gaze shift conditions. Multiple comparisons were corrected by a non‐parametric approach via a Monte Carlo method (Maris & Oostenveld, 2007). A reference distribution of t‐values was approximated by 5000 random permutations of the theta or alpha relative power values corresponding to both the Congruent and Incongruent conditions and the ROIs. By doing so, a histogram of the test statistics was obtained, and the 95% alpha level could be calculated.

To control that the differences between the Congruent and Incongruent gaze shift conditions were not due to accidental differences of the band power in the baseline periods, the theta and alpha values in the baseline period of the Congruent and Incongruent object‐gaze shift conditions were compared via a paired *t* test.

## 3 RESULTS

No significant differences in the number of trials used for the TFR calculation were observed between the Congruent and Incongruent object‐gaze shift conditions [respectively, median (min‐max): 11 (8–23), 11 (8–26), Mann‐Whitney test *p* = .848]. The mean across subjects (± standard deviation) of the interval elapsed between the direct gaze onset and the object presentation was 1.187 ± 0.245 ms. The mean across subjects (± standard deviation) of the coefficient of variation of these intervals (defined as the standard deviation over the mean in the single subject) was 0.24 ± 0.09.

No significant differences were found between Congruent and Incongruent conditions for the intervals elapsed from the object presentation to the averted gaze onset (respectively 1.175 ± 0.203 s and 1.167 ± 0.173 s, two‐tailed paired *t* test: *t*[16] = 0.645, *p* = .128). Similarly, no significant differences were found between the coefficients of variation [0.16 ± 0.08 and 0.17 ± 0.08, respectively for Congruent and Incongruent condition, two‐tailed paired *t* test: *t*(16) = −0.719, *p* = .483].

**FIGURE 3:**
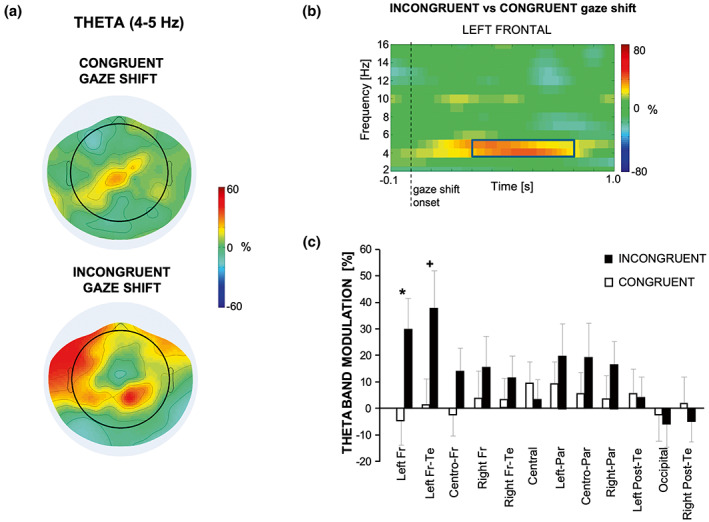
(a) Scalp topography of the theta power modulation (ERD/ERS) in the Congruent and Incongruent object‐gaze shift conditions during the 300–800 ms interval after the gaze shift. (b) Average time‐frequency representation of the relative power in the left frontal area of the difference between Congruent and Incongruent object‐gaze shift conditions. The black box indicates the frequency band considered for theta band power computation and the time interval used for comparing the Congruent and Incongruent object‐gaze shift conditions. (c) Means across subjects and standard errors of the power in the theta band for the Congruent (white) and Incongruent (black) object‐gaze shift conditions in each of the 12 ROIs. Star and cross indicate significance of paired *t* test (corrected *p < .05, ^+^ *p* = .12)

Figure 3a shows the scalp topography of the theta band modulation in the Congruent and Incongruent object‐gaze shift conditions during the interval of 300–800 ms after the gaze shift onset (Figure 3b). Repeated measures ANOVA on the theta band power modulation showed a significant main effect of *ROI* [*F*(11, 176) = 2.31; *p* = .012; $\eta_{P}^{2}$ = 0.126] as well as a significant *Condition* × *ROI* interaction effect [*F*(11, 176) = 2.61; *p* = .004; $\eta_{P}^{2}$ = 0.140], but no significant main effect of *Condition* (*p* = .116). Two tailed paired sample *t* test applied separately to each ROI yielded significant differences, with incongruent greater than congruent theta power modulation values for the left frontal region [*t*(16) = 3.12; *p* = .006, corrected *p* = .010; Figure 3c]. A similar trend was also found for theta power in the left fronto‐temporal region [*t*(16) = 1.83; *p* = .086, corrected *p* = .120; Figure 3c].

**FIGURE 4:**
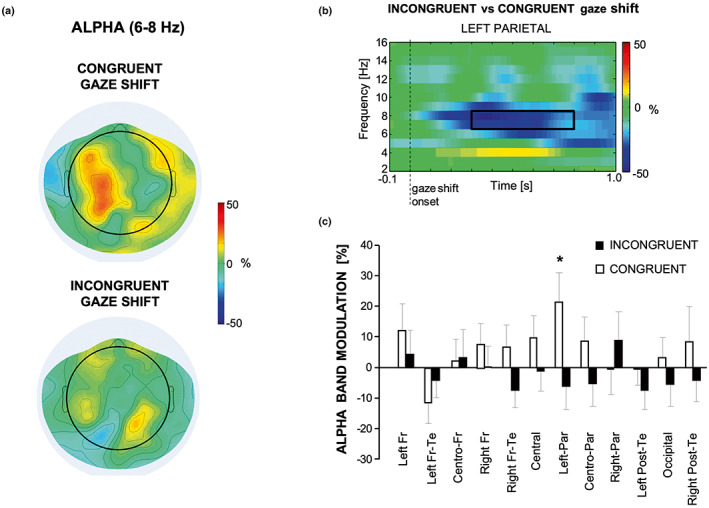
(a) Scalp topography of the alpha power modulation (ERD/ERS) in the Congruent and Incongruent object‐gaze shift conditions during the 300–800 ms interval after the gaze shift. (b) Average time‐frequency representation of the relative power in the left parietal area of the difference between Congruent and Incongruent object‐gaze shift conditions. The black box indicates the frequency band considered for alpha band power computation and the time interval used for comparing the Congruent and Incongruent object‐gaze shift conditions. (c) Mean across subjects and standard errors of the power in the alpha band for the Congruent (white) and Incongruent (black) object‐gaze shift conditions in each of the 12 ROIs. Star indicates significance of paired *t* test (corrected *p < .05)

Figure 4a shows the scalp topography of the alpha band modulation in the Congruent and Incongruent object‐gaze shift conditions during the interval of 300–800 ms after the gaze shift (Figure 4b). Repeated measures ANOVA on the alpha band power modulation showed only a significant *Condition* × *ROI* interaction [*F*(11, 176) = 2.14; *p* = .020; $\eta_{P}^{2}$ = 0.118], but no significant main effect of ROI (*p* = .158) or Condition (*p* = .359). Two‐tailed, paired‐sample, *t* test applied separately to each ROI yielded significant differences for the alpha power in the left parietal region, with congruent greater than incongruent alpha modulation values [*t*(16) = −2.38, *p* = .030, corrected *p* = .050; Figure 4c].

The differences found between the Congruent and Incongruent conditions were not due to different values of theta and alpha bands in the baseline period, as evidenced by the paired *t* test between band power values of the Congruent and the Incongruent gaze shift conditions (*p* > .2 for both bands).

## 4 DISCUSSION

The current study examined the modulation of brain activity in 9‐month‐old infants in association with the use of gaze as a referential cue within triadic interactions. Consistently with previous studies (Hoehl et al., 2008; Michel et al., 2015; Rayson et al., 2019; Senju et al., 2006), we found infant brain activations related to gaze processing in the theta and alpha bands. Differently from these studies, that used computerized stimuli, we employed a close‐to‐natural live paradigm, which, despite the relatively small number of infants included in the study, permitted to obtain results that have a higher ecological validity.

The theta power increase observed in the left frontal and fronto‐temporal regions under the Incongruent versus Congruent gaze shift condition, which could be related to oculo‐muscular activity, could also be explained by the mechanism of violation of expectation (Berger et al., 2006; Köster et al., 2019, 2021). Infants develop a basic understanding of their physical (Baillargeon et al., 1985; Spelke et al., 1992) and social (Reid et al., 2009) surrounding by the first few months of age. Köster et al. (2019, 2021) suggested a mechanism of “violation of expectation” for human actions to explain the theta power increase observed in 9‐month‐old infants in association with unexpected outcomes: events that violate the infants' basic expectations elicit a prediction error and thus require the infants to modify their predictions, with a consequent theta rhythm modulation during the refinement of the initial environment representations. The direct gaze and smiling that the adult and the infant exchange at the beginning of each trial represent powerful non‐verbal cues commonly used with infants to communicate that they are addressed for a social exchange. Therefore, when the communicative adult produces an eye gaze shift following the appearance of an object, the infants likely expect that the gaze shift would be toward the object, as it occurs in real episodes of JA (Csibra & Gergely, 2009; George & Conty, 2008; Kleinke, 1986). Our close‐to‐natural paradigm could have contributed to strengthen the infant's prediction of the adult's gaze as object directed. Therefore, when the adult turns her eyes away from the presented object, the situation could have been perceived as socially “bizarre” and violating the infants' initial prediction, thus increasing their attention and possibly enhancing the theta activity.

Notably, the increase of the theta activity during the Incongruent gaze‐shift condition was localized in the frontal brain areas. Theta band activity in these regions is typically related to the involvement of the executive attention network, responsible for the control of internal attentional processes (Cavanagh & Frank, 2014; Kam et al., 2019; Orekhova et al., 1999, 2006). Executive control of attention emerges gradually during the first year of life: differently from involuntary attention, which is automatically driven by surface characteristics of the stimulus during the first 4 months of life, an executive attention network matures from 6 months of age onward (Bazhenova et al., 2007; Orekhova et al., 1999, 2006). Accordingly, Orekhova et al. (1999) found higher theta oscillations over the frontal and temporal brain areas in infants aged 8–11 months who maintained the internally controlled attention for a relatively long period of time. Furthermore, behavioral studies on JA abilities evidenced that 9‐month‐old infants are increasingly able to detect their own and another person's attention, depending on volitionally controlled shifts of attention (Mundy & Newell, 2007). Our findings seem to be in agreement with prior studies on gaze cueing processing (Bazhenova et al., 2007; Begus & Bonawitz, 2020; Michel et al., 2015) supporting the notion that a maturation of the executive control of attention occurs in the second half of the first year of life. The advancement of their cognitive abilities would allow the infants not only to process the object‐gaze relationship but also to specifically follow the social partners' gaze.

We also found a left lateralization of the theta power enhancement in the frontal brain areas. Similarly, NIRS studies of Grossmann and Johnson (2010), Grossmann et al. (2013) showed that 5‐month‐old infants recruited regions localized in the left dorsal prefrontal cortex during triadic social interactions. Similar adult studies confirmed the involvement of the left medial dorsal prefrontal cortex in the reward‐related brain network (Schilbach et al., 2010), responsible for motivation to approach environmental stimuli (Fox, 1991). In agreement with speculations by other authors (Bates, 1979; Mundy & Jarrold, 2010; Woodward, 2003), our results support the idea that neural specialization for social cognition involving the left prefrontal cortex might emerge before the end of the first year of life, when infants begin to detect and follow the gaze of their social partner.

Differently from recent research (Hoehl et al., 2014; Michel et al., 2015; Rayson et al., 2019), we found an increased alpha activity during Congruent versus Incongruent object‐gaze shifts. According to the literature (Pfurtscheller et al., 1996; Steriade & Llinás, 1988), alpha desynchronization is related to information processing, whereas synchronized alpha activity reflects a kind of “iding state” of cortical activity. However, some studies revealed that, under specific experimental conditions, local alpha synchronization can be maximal during highest task demands, suggesting that a modulation of the alpha band activity could be also related to the inhibition of interfering, task irrelevant information (Fernández et al., 1998; Klimesch et al., 1999). Support to this “inhibition‐timing hypothesis” (Klimesch et al., 2007) comes also from developmental research. In 8‐ to 11‐month‐old infants, Orekhova et al. (2001) found a higher alpha synchronization over the posterior parietal region during an anticipatory attention task, and associated this finding with the active inhibition of irrelevant peripheral information to avoid interference of concurrent visual stimulation. This mechanism is essential for the emergence of top‐down processes such as the internally controlled attention and can develop over the second half of the first year of life, when an executive and voluntary attention, regulated by intentions and task demands, emerges. Our live paradigm could have enhanced the infants' attention in the Congruent condition. In fact, it reproduces, although basically, real‐life JA episodes, where the adult looks at and selects a particular object in the environment. Therefore, the infant perceives this object as a special target, and pays attention to it in order to capture new incoming meanings. To specify, infants involved in our Congruent object‐gaze shift condition had to maintain their attention on the cued object, as the expected result of the adult behavior.

## 5 CONCLUSION

To our knowledge, our study is the first that investigated alpha and theta oscillations in 9‐month‐old infants engaged in a live paradigm suited to measure gaze cueing effects. In agreement with previous studies, our finding on the theta power increase in response to the adult object averted gaze could reflect the infant response to an unexpected event, representing a marker of information prediction about a common social behavior. Our live paradigm, inducing the infants to expect what usually happens in everyday social encounters, that is, that the adult's eyes would be directed to the object just presented instead of elsewhere, might have enhanced the infant response to the Incongruent condition.

We also found a greater alpha power in the Congruent condition. This result contradicts some prior studies but is consistent with few other studies supporting the notion that a higher alpha power occurs in infants in relation to the inhibition of interfering stimuli needed to sustain an internally controlled attention. Our live paradigm, favoring the expectation of a usual event, could have enhanced this effect.

Our study has some limitations that need to be addressed in future research. First, we explored the infant's brain responses at 9 months of age. However, previous neural studies using computerized stimuli (Grossmann et al., 2013; Rayson et al., 2019) revealed a more precocious sensitivity of infants in encoding the gaze‐object relation. Future studies should include infants at younger ages to investigate whether a more interactive and engaging context as a live paradigm could promote an earlier modulation of the theta and alpha band related to eye gaze cuing. Second, given that an impairment in JA abilities like the eye gaze processing is supposed to characterize children affected by the ASD (Elsabbagh et al., 2012; Gillespie‐Lynch et al., 2013), and considering that computerized setups are only an approximation of real‐life conditions, it would be interesting to use a live paradigm reproducing daily experiences of ASD children to deepen the understanding of the mechanisms underlying the atypical development of JA abilities in an ASD population (Baron‐Cohen et al., 1995; Frith & Frith, 2001).

Finally, we used only a live setup. Future research should compare live face‐to‐face versus standard PC paradigms to evaluate the supposed “live paradigm effect,” that is, the impact of the different levels of interaction implemented in the experimental setup on the assessment of neural activations, despite the challenging technical issues associated with live paradigms and the high drop‐out rates that can be predicted.

## AUTHOR CONTRIBUTIONS

**Laura Angelini:** Conceptualization; data curation; investigation; methodology; validation; writing – original draft; writing – review and editing. **Gabriella Tamburro:** Data curation; formal analysis; investigation; methodology; software; validation; writing – original draft; writing – review and editing. **Francesca Lionetti:** Writing – review and editing. **Maria Spinelli:** Writing – review and editing. **Silvia Comani:** Supervision; writing – original draft; writing – review and editing. **Filippo Zappasodi:** Data curation; formal analysis; software; writing – original draft; writing – review and editing. **Mirco Fasolo:** Supervision; writing – original draft; writing – review and editing. **Tiziana Aureli:** Supervision; writing – original draft; writing – review and editing.

## CONFLICT OF INTEREST

The authors declare that the research was conducted in the absence of any commercial or financial relationships that could be construed as a potential conflict of interest.

## Data Availability

The data supporting the findings described in this article will be available from the authors upon reasonable request.
